# Barriers and facilitators of pharmacists’ integration in a multidisciplinary home care team: a qualitative interview study based on the normalization process theory

**DOI:** 10.1186/s12913-024-11014-y

**Published:** 2024-05-02

**Authors:** Karl-Erik Bø, Kjell H. Halvorsen, Anna Yen-Ngoc Le, Elin C. Lehnbom

**Affiliations:** 1 Department of Pharmacy, Faculty of Health Sciences, UiT The Arctic University of Norway, Tromsø, N-9037 Norway; 2 Department of Health and Caring Sciences, Faculty of Health and Life Sciences, Linnaeus University, Hus Vita, Kalmar, 431 26 Sweden

**Keywords:** Qualitative, Pharmacist, Implementation, Home care, Normalization process theory

## Abstract

**Background:**

There is a growing recognition of multidisciplinary practices as the most rational approach to providing better and more efficient healthcare services. Pharmacists are increasingly integrated into primary care teams, but there is no universal approach to implementing pharmacist services across healthcare settings. In Norway, most pharmacists work in pharmacies, with very few employed outside this traditional setting. The home care workforce is primarily made up of nurses, assistant nurses, and healthcare assistants. General practitioners (GPs) are not based in the same location as home care staff. This study utilized the Normalization Process Theory (NPT) to conduct a process evaluation of the integration of pharmacists in a Norwegian home care setting. Our aim was to identify barriers and facilitators to optimal utilization of pharmacist services within a multidisciplinary team.

**Methods:**

Semi-structured interviews (*n* = 9) were conducted with home care unit leaders, ward managers, registered nurses, and pharmacists in Norway, in November 2022-February 2023. Constructs from the NPT were applied to qualitative data.

**Results:**

Findings from this study pertain to the four constructs of the NPT. Healthcare professionals struggled to conceptualize the pharmacists’ competencies and there were no collectively agreed-upon objectives of the intervention. Consequently, some participants questioned the necessity of pharmacist integration. Further, participants reported conflicting preferences regarding how to best utilize medication-optimizing services in everyday work. A lack of stakeholder empowerment was reported across all participants. Moreover, home care unit leaders and managers reported being uninformed of their roles and responsibilities related to the implementation process. However, the presence of pharmacists and their services were well received in the setting. Moreover, participants reported that pharmacists’ contributions positively impacted the multidisciplinary practice.

**Conclusion:**

Introducing new work methods into clinical practice is a complex task that demands expertise in implementation. Using the NTP model helped pinpoint factors that affect how pharmacists’ skills are utilized in a home care setting. Insights from this study can inform the development of tailored implementation strategies to improve pharmacist integration in a multidisciplinary team.

**Supplementary Information:**

The online version contains supplementary material available at 10.1186/s12913-024-11014-y.

## Background

Primary care services are acknowledged as crucial for promoting the overall well-being of individuals and communities [1]. Serving as the initial point of contact for people with chronic illness, they are often regarded as the backbone of health systems in many countries. Offering holistic and continuous care for patients and their families, accessible and functional primary health services can help reduce the burden of avoidable illness and injury.

It is well established from a variety of studies that primary care services are under severe pressure. The causes of this emerging crisis are multifaceted, with the aging population, growing clinical complexities, and workforce shortages being primary contributors [2]. Addressing these issues necessitates an investment in professional development, and multidisciplinary practices are recognized as a rational approach to better and more efficient patient care. However, ensuring optimal use of human resources and competencies while maintaining the coverage and reach of high-quality services requires a redistribution of labor among healthcare workers.

There is growing recognition of the beneficial contributions pharmacists can make in addressing healthcare challenges and improving patient outcomes [3, 4]. The global challenges of polypharmacy and co-morbidity have expanded pharmacists’ scope of practice beyond drug dispensing and countries like the US, Canada, the UK, and Australia have made substantial advancements in utilizing pharmacists’ competencies in primary care settings [5]. However, integrating new professions within multidisciplinary teams is a complicated process and evidence suggests that optimal utilization of newly embedded competencies is prone to several barriers [6, 7].

The success of a new program or an intervention is contingent not only on its inherent efficacy but also on the engagement and buy-in of stakeholders, alignment with existing workflows, and the capacity of the system to support change. During the early phases of implementation, staff are generally supportive and committed to a new program. This optimism is partly driven by the anticipation of positive outcomes. However, the chance of relapse or failure is considerable as the day-to-day challenges of implementation become apparent [6].

Sustainability, which refers to the continuation of programs and behaviors beyond the initial stages of adoption, presents a significant challenge in implementation practice [8]. A growing body of literature reflects the active and important role of context in this process [9]. Consequently, recurring evaluations are pivotal to maintaining the benefits of an intervention; analyzing team performance reveals whether change is under way and improvement is recognized [10]. These assessments are important for each new program as implementation strategies are likely to be more effective when they are customized to specific determinants [6].

This study set out to perform a process evaluation of the integration of pharmacists in a Norwegian home care setting. Our aim was to identify barriers and facilitators to optimal utilization of pharmacist services within a multidisciplinary team.

## Methods

### Norwegian primary care

In Norway, municipalities are at the lowest level of public administration [11]. They are responsible for managing and providing primary care as part of the public health system. Norwegian primary care comprises services such as general practitioners (GP), nursing homes, and home care.

### Norwegian home care services

Home care enterprises in Norway provide health and social care services to people who live in their own home or in residencies within a community. The comprehensive process of coordinating, optimizing, and dispensing medications is a major part of these services. Norwegian home care settings are typically organized in the following manners: [12]

1. Community dwellings with a co-located staff (> 50%).
2. Ambulatory services provided to people living in their own homes (15%).
3. A combination of 1 and 2. (14%)

In contrast to nursing homes, physicians are not co-located with the staff base in home care settings. Each home care patient attends to a general physician (GP) of their own choice. Even though GPs play an instrumental role in securing the appropriate use of health services for local communities, human resources in Norwegian home care settings comprise mainly registered nurses, assistant nurses, and assistant healthcare workers.

### Health workforce shortages

Like other European countries, Norway is experiencing a severe shortage of nursing personnel, and statistical models predict an under-coverage of 13.000 registered nurses by 2030 [13]. To further complicate this issue, municipalities grapple with a crisis in GP recruitment [14].

### Characteristics of pharmacists working in Norway

Norwegian pharmacists work predominantly in community pharmacies. Community pharmacies are owned by three major international pharmacy chains, each of which is vertically integrated with a pharmaceutical wholesaler. These pharmacies operate as highly commercial businesses and are standalone entities separated from both nursing homes and home care settings. Norwegian pharmacists are not authorized to (de)prescribe medications.

Despite Norwegian health authorities advocating for more focus on reducing medication errors in primary care, there is no national policy for integrating pharmacists in these settings. There have been scattered and moderately successful initiatives to implement pharmacist services within both nursing homes and home care settings [15, 16]. These novel initiatives have been driven by local stakeholders and innovative municipalities.

### The normalization process theory (NPT)

The NPT was developed to address the difficulties of implementing complex interventions in healthcare settings [17]. It is concerned with practical problem-solving at the micro-levels of an organization.

May and colleagues describe normalization as *“the embedding of (…) an organizational change as a routine and taken-for-granted element of clinical practice”* [18]. The NPT views the process of embedding and integration of these changes as the contingency of work (implementation). As such, the way work is produced and organized in a setting will affect whether a practice becomes integrated into daily routines. Drawing on findings from empirical studies, the NPT proposes four determinants that influence the embedding of a new practice. Table 1 outlines this study’s operationalization of the NPTs determinants as described by May et al [17, 19, 20]. All constructs and sub-constructs are not covered equally in our analysis but the table gives a comprehensive overview of relevant implementation aspects that guided the interpretation of our data.

**Table 1:** operationalization of NPT constructs as derived from May et al. [17, 19, 20]

*Coherence*: How do participants understand and make sense of the new work methods?	*Cognitive participation*: How do participants commit to and engage in the new work methods?	*Collective action*: How are participants organized to facilitate the enacting of the new work methods?	*Reflexive monitoring*: How do participants appraise and reflect on the new work methods?
*Differentiation*: Do the actors perceive the pharmacist services as innovative?	*Initiation*: How are actors motivated to implement pharmacist services?	*Interactional workability*: What is the role of each participant in interacting with the pharmacist services?	*Individual appraisal*: How do actors assess the value and effectiveness of pharmacist services?
*Individual specification*: How do stakeholders conceptualize pharmacist services?	*Enrolment*: How are actors organized to participate in the new work practice?	*Relational integration*: Does the team have the required knowledge to utilize the pharmacist’s services?	*Systemization*: What rationalities underpin the judgments of the pharmacist services (informal/formal)
*Communal specification*: Is there a shared understanding of the objectives of the pharmacist services?	*Legitimation*: How do stakeholders achieve ‘buy-in’ for the pharmacist services?	*Skill-set workability*: How are actors trained and organized to implement pharmacist services?	*Communal appraisal*: How do stakeholders collectively judge the value and effectiveness of pharmacist services?
*Internalization*: Do stakeholders understand the potential and value of pharmacist services?	*Activation*: How are pharmacists’ work methods effectively operationalized within the home care setting?	*Contextual integration*: How are resources organized and allocated to support the integration of pharmacist services?	*Reconfiguration*: How are the pharmacist services modified and reconstructed based on evaluations?

### The concept of integration

The term integration can be operationalized in diverse ways. Walshe and Smith provide a conceptual framework for assessing the degree of integration into a team related to the ‘harder’ aspects of work, such as job fraction, and access to information systems [21]. This approach to integration assessment is less suited for our study as the pharmacists in our research are full-time salaried, on-site workers.

The NPT relates the terms embedding and integration to the ‘softer’ dimensions of an organization such as the interplay between the practice itself and individuals in the social environment in which the implementation takes place. Our research pertains to the definition of integration as a result of successful implementation work, i.e., *“the social process of bringing a practice into action.”* [22].

### Objectives

This study collected qualitative data to answer research questions related to an organization’s readiness to utilize the pharmacists’ competencies:

- What is the non-pharmacist healthcare professional’s knowledge, beliefs, and expectations of the pharmacists and the pharmacist services?
- What are the pharmacists’ expectations and experiences using their competencies within a home care setting?
- How do leaders engage and organize team members in integrating a new profession and work methods?

### Study design

This was a qualitative interview study designed to inform on implementation strategies to integrate pharmacists into Norwegian home care settings. It was the follow-up of a quality improvement project conducted in a similar setting [16].

The interview questions were developed and inspired by the theoretical constructs in an extended version of the NPT [23]. Questions in the interview guide were related to topics such as expectations to pharmacist services and perceptions of the implementation process. The interviewer (AL), supervised by a fellow researcher (KHH), piloted the interview guide for clarity and training purposes. No guide changes were made following the piloting. The interview guide is provided in Appendix 1.

### The research team and reflexivity

The research team included two female pharmacists (AL and ECL), and two male pharmacists (KEB and KHH). The first author (KEB) was a Ph.D. student with experience in managing community pharmacies. AL was a Master of Pharmacy student with work experience in community pharmacies. The rest of the research team (ECL and KHH) were associate professors and had backgrounds in health services research. Three of the authors (KEB, KHH and ECL) had knowledge of and experience in qualitative methodology and qualitative interview research.

All authors were familiar with the healthcare system and settings in which the research was performed.

### The research setting

The setting researched in this study was part of a home healthcare organization covering several city boroughs in one of the larger cities in Norway. The home care organization consisted of separate units, each made up of multiple home care wards. Each home care unit was managed by a unit leader, and each ward was led by ward managers.

The research setting decided to hire on-site pharmacists in permanent positions based on an internal evaluation conducted during a two-year pilot phase. This pilot was conducted within the same home care organization, but in a separate unit, and aimed to enhance patient safety by integrating pharmacist services. Although the evaluation provided some anecdotal insights, its limited scope may not have fully captured the complexity of the implementation process. Further, the integration approach during the pilot was pragmatic, resembling what some researchers refer to as *“letting it happen,”* where a process unfolds organically without extensive planning [24].

### The intervention

At the time of the data collection, the pharmacists in the setting provided a wide range of services targeting patients, healthcare personnel, and intern students. Some of the more frequently provided services were medication therapy optimizing services such as medicines reconciliation and medication reviews, and health personnel education. The pharmacists did not have any job description, nor were they given any clinical training in advance of their introduction to the home care setting. Their experience levels differed; some had less than a year of experience in the research setting, while others had been working there for two years.

### Recruitment and data collection

During the early stages of the research, one of the authors performed visits to units in the home care setting (AL). The purpose of these visits was to invite a strategic sample of informants to attend the research. The recruitment process was completed via e-mail.

Data was collected from one home care unit which consisted of four home care wards. A total of nine interviews were conducted. All interviews were carried out by one researcher (AL), except for one that was carried out by two researchers (AL and KEB). The participants were: registered nurses (2), unit leader (1), ward managers (4), and pharmacists (2). All data was collected at the home care workplace except one interview that was conducted at the university campus. Non-participants were not present. The consent form held information on the main objectives of the research.

The leaders and managers participating in this study were healthcare personnel and formally appointed “mid-level leaders,” i.e., individuals who supervise others and manage home care units or wards through a moderate level of authority. For simplicity, both unit leaders and ward managers will be collectively referred to as “home care leaders” in this paper.

Semi-structured interviews were carried out in November 2022 and February 2023. Each interview lasted for 25–70 min and was audiotaped. Field notes were made after each interview. All interviews were transcribed verbatim by two researchers (AL and KEB). All participants received a ‘thank you’ voucher worth £50 upon completion of the interview.

### Data triangulation

Aiming to investigate the home care teams readiness to integrate pharmacists as new members of their collaborative practice, our focus was to interview home care leaders. These individuals are recognized to be key links between the strategic decisions made by program planners and the healthcare professionals who must interact with the pharmacists in everyday work. To achieve a broader understanding of the combined knowledge on the implementation process we decided to include pharmacists and registers nurses.

### Data analysis and reporting

Two researchers (AL and KEB) analyzed the entire data corpus separately. The analysis was inspired by a thematic ‘bottom-up’ approach, i.e., aiming to provide a comprehensive analysis starting at a low level of abstraction. Meaning units relevant to our research questions were identified using a combined approach of inductive data-driven coding and deductive interpretation. Condensed meaning units were coded within each interview and the most relevant codes for our research questions were abstracted and clustered into themes. This process was iterative and conducted for each interview. Transcripts and audiotaped recordings were revisited several times during the research process. Member-checking was not applied in this study.

As the analytical process evolved, the level of abstraction progressed from descriptive to interpretive. At this stage of analysis, identified themes were discussed regularly with co-authors. Ultimately, the results of each interview were compared and combined in a cross-sectional analysis. In the final stages of the analysis, constructs from the NPT guided the clustering of codes into themes. MindManager™ software was used to organize codes and themes during the analysis.

The research was guided by the consolidated Criteria for reporting qualitative research (COREQ) [25].

## Results

### Coherence

#### How do participants understand and make sense of the new work methods?

Nurses and home care leaders reported a lack of experience working with pharmacists and were mostly unfamiliar with the pharmacists’ skill set. Despite this limited interaction and knowledge, most informants envisioned pharmacists as having distinct and advanced expertise in medication management, potentially exceeding that of nurses and physicians. When asked to elaborate on this statement, one of the leaders found it difficult to point to any specific skills:

*“I don’t know how to explain it. It is too advanced; it is beyond my abilities to address this.” (Home care leader)*.

The participants in this study had different views on medication challenges within the home care setting. Home care leaders and registered nurses expected pharmacists to take on tasks usually performed by nursing staff, such as ‘double checking’ dispensed tablets, believing it would allow more time for nursing. This expectation was consistently highlighted by every non-pharmacist interviewee. In contrast, pharmacists showed a preference for patient-focused activities such as medication reviews and reconciling medication lists. While pharmacists were open to new roles, they were reluctant to engage in work they considered more appropriate for other health professions. They also had concerns about the expected volume of non-clinical work. Reflecting on these incompatible expectations, one pharmacist reported having difficulties balancing being helpful with doing meaningful work:

*“It has been a challenge. There are just so many medication-related issues, and I find it difficult to figure out where to engage, and what to prioritize.” (Pharmacist)*.

Home care leaders and nurses commonly expressed their expectations of pharmacist services by using the term “quality assurance”. This phrase suggests an anticipation that pharmacists would enhance all facets of medication work. Pharmacists reported being familiar with the use of this expression and commented on its vagueness:

*“They expect us to improve the quality of medication work. But how? They leave it to us to figure that out.” (Pharmacist)*.

### Cognitive participation

#### How do participants commit to and engage in the new work methods?

Home care leaders commonly reported detachment from the decision-making process regarding the introduction of pharmacists in the home care team. Additionally, they knew little of the pharmacist recruitment procedure which led to a lack of engagement in facilitating integration. They perceived the task of incorporating pharmacists into the home care team as outside their remit, attributing the responsibility for the project to higher-level authorities. This view of pharmacist integration as an externally imposed initiative was consistent among all participants, highlighting a disconnect between decision-makers and the expected implementers:

*“The decision is probably made by someone at the town hall” (Home care leader)*.

And:

*“I had no say in this process, we were merely informed about the decision.” (Home care leader)*.

Additional confusion arose from differing views on whether pharmacist roles were temporary for a pilot or permanent. Furthermore, several home care leaders did not perceive the pharmacists as integrated team members but rather as external to their workforce.

In general, the participants in this study called for more empowerment as they reported having little knowledge regarding the implementation process. Moreover, most home care leaders reported that the lack of instructions and guidance caused them to be insecure about their roles and responsibilities in the project. One leader explicitly stated that the implementation process was confusing and that it made it difficult to know how to effectively utilize the pharmacists in everyday work:

*“I find it difficult to address how we can utilize the pharmacists’ competencies mainly because of the vague implementation process. I imagine that pharmacists, as a profession, have comprehensive skills but I am clueless about what they do.” (Home care leader)*.

Interviewed pharmacists described the implementation process as unclear and found adapting to the new work environment confusing. Some of this insecurity was related to the lack of job-descriptions and standard operating procedures. Non-pharmacist interviewees also expressed uncertainty regarding the pharmacist’s job description. Some home care leaders believed a job description might exist within the organization, and that access to this document would have facilitated pharmacist integration. Even though other participants doubted its existence, a pharmacist confirmed that a job description was available and should be accessible to all home care leaders electronically.

### Collective action

#### How are participants organized to facilitate the enacting of the new work methods?

Workforce shortage was reported to be a challenge in the process of integrating the pharmacist into the home care team. The situation of low staffing was perceived as a permanent issue caused by a high personnel turnover and nursing shortages. Several informants stated that the high workload of both nurses and home care leaders made it difficult to include pharmacists in the teams’ daily routines. One participant expressed concern that the integration of pharmacists would introduce additional time-consuming procedures, intensifying the strain on already limited resources. Others reported that compared to monodisciplinary work, multidisciplinary work was more demanding. Consequently, they felt that low staffing made collaboration with the pharmacists difficult:

*“Collaborating with new or inexperienced colleagues is very time consuming. In situations of low professional staffing, it is often impossible to prioritize engaging in multidisciplinary work with the pharmacist” (Registered nurse)*.

A challenge reported by most participants was the perceived inequality of access to pharmacist services across the four separate home care wards. Even though the pharmacists were located on-site, the total number of pharmacist positions in the home care unit was not sufficient to cover all wards equally. Consequently, health personnel working at wards co-located with the pharmacist’s office reported to have the easiest access to pharmacist services. The pharmacists perceived this situation as worrying as they felt obligated to provide an even number of services across each ward. Moreover, non-pharmacist interviewees reported feeling that they were treated inequitable and that they missed the opportunity to receive medication-optimizing services:

*“It would be great to be better acquainted with the pharmacists, but we [a specific ward] feel a bit detached from this initiative and the pharmacist services.” (Home care leader)*.

The perceptions of the pharmacists’ role and position in the home care team varied among the participants. However, the interviewed home care leaders were undivided in the perception that the main responsibility for utilizing pharmacist competencies lay with the pharmacists and that they had to rise to the occasion. This assumption was mirrored in the perceptions among the pharmacists as they reported sensing these expectations and feeling the need to prove themselves worthy of a position in the home care setting.

### ’’Reflexive monitoring

#### How do participants appraise and reflect on the new work methods?

Participants generally welcomed the integration of pharmacists into their teams, often linking their positivity to potential relief in staffing challenges and nursing shortages. Further, they anticipated delegating medication responsibilities to their new colleagues. One participant humorously compared pharmacists to professors, highlighting their academic capabilities. However, most participants saw pharmacists mainly as consultants for medication inquiries. When specifying the types of medication-related questions that commonly arose, some participants realized that many of these queries were trivial and could be effortlessly answered with a few clicks on a computer.

Although the pharmacists had been working in the home care environment for approximately seven months, most participants reported neither recognizing nor observing any visible changes to the medication-related activities in the setting. One home care leader responded not knowing whether anything had changed, yet another reported that everything remained the way it always had been. However, when asked more specifically about their experiences with the pharmacists, most managers and leaders responded that pharmacists were just recently employed in the setting and that it was too soon to draw any conclusions regarding their performance. Still, they did not hesitate to describe the integration of pharmacists in their home care team as successful. One home care leader explicitly stated that the implementation process was effortless and straightforward.

Despite having very limited knowledge of both the objectives of the implementation process and the scope of the pharmacist services, several participants expressed a predetermined belief in the necessity of the pharmacist integration. A couple of the informants reported that there was a collective decision to support the services regardless of any evidence of effectiveness. The same informants stated there was a joint agenda to persuade decision-makers to scale up the initiative of integrating pharmacists into primary care:

*“The idea, from the very beginning, was that these services should be integrated into all home care wards. People do their best to influence the decision-makers in higher positions of authority” (Home care leader)*.

And:

*“As long as the higher-level individuals of the organization are convinced and on board with the idea, we are all good” (Home care leader)*.

## Discussion

This paper applied the NPT to identify aspects of the implementation process that can enable or hinder the integration of pharmacist services within a Norwegian home care setting. Even though some of the constructs in this theory are flexible and open to interpretation, it is considered a comprehensive and robust guide to implementation [20].

### How do participants understand and make sense of the new work methods?

The NPT states that implementation is influenced by factors that promote or hinder actors’ sense-making of a practice. Consequently, understanding the acts and behaviors that make up this practice is a reasonable starting point for the assessment of an implementation process [26].

In our study, non-pharmacist interviewees seemed to have limited knowledge of the pharmacists’ skill levels and difficulties conceptualizing the pharmacist services in detail. Similar challenges are described in a systematic review from 2020 in which Hatton and colleagues reported that a lack of knowledge of the pharmacist role led to misconceptions and consequently hindered integration [27].

Working mainly with compounding and dispensing activities, pharmacists have traditionally focused on their own role in isolation from other health professions. As pharmacists increasingly become part of multidisciplinary teams, they may face challenges due to a history of working separately from nurses and physicians. Further, pharmacist services are complex. They have a high degree of flexibility and can be directed toward diverse groups of stakeholders (e.g., patients, colleagues, and organizations) to impact different outcomes [28]. Lacking a clear description of the intervention and precise definitions of its activities can hinder its usability [29]. Even services such as the medicines reconciliation and the medication review, which appear conceptually uncomplicated, comprise both sub-interventions and several elements of multidisciplinary work [30].

### Engagement of stakeholders in the program

According to the NPT, the integration of new work methods within a team depends on efforts to organize the actors and activities implicated in a practice. To support the introduction of new work methods in healthcare it is necessary for program planners to systematically assess whether the intervention suits the organization’s needs [24, 31]. Moreover, early and widespread staff involvement can clarify key elements of the implementation and increase commitment to the process [32].

In our study, home care leaders reported having very little knowledge regarding the rationale and objectives of the pharmacist integration program. They expressed being unaware of the reasons why their wards were provided with a new profession and reported not being empowered in this decision. This knowledge gap and the lack of agreed upon objectives resulted in conflicting expectations within the team.

Team management and facilitation are the leader’s responsibility. Thus, characteristics of leaders and leadership within an organization can be critical to the improvement and implementation of primary care initiatives [33]. In their research from 2014, Aarons and colleagues identified four dimensions of leadership behavior to support implementation [31]. Two of these dimensions were related to the leaders’ knowledge about the practice or innovation, and to being proactive in anticipating implementation issues, respectively. Further, role clarification is recognized as a salient aspect of successful collaborative practices. It is difficult to commit to the implementation process without knowing your formal responsibility, and ambiguities related to the roles of healthcare providers can lead to workplace tension and underutilization of professional expertise [34]. The importance of professional role clarity and identity has been reported in several pharmacist integration initiatives [35–37].

The NPT highlights that it is important to consider how new work methods interact with already existing practices. Consequently, a multidisciplinary team needs to have a shared understanding of the objectives of new practices. However, program planners often view an intervention in isolation from the overall system. A recent UK study illustrates how this tendency to underestimate the complexity of healthcare settings can cause unnecessary pauses and recalibrations of improvement programs [38].

Teams within a healthcare organization often have their habitual routines and work processes. Organizational routines comprise a mix of coordinated and recurrent behavior patterns which reduce the uncertainty and complexity of individual decisions [39]. As such, new and more complex interventions can be conceived as attempts to disrupt existing system dynamics. Our findings indicate that intensive medication tasks, such as medicines reconciliation and medication reviews, were undervalued in the setting. Prior to pharmacist integration, quality reports revealed that only about 10% of patients underwent medication reconciliation. The introduction of this task, now performed more frequently, may hamper established workflows for non-pharmacists. Despite the municipality’s and pharmacists’ goals to execute these demanding services, nurses and home care leaders appeared to consider them less essential to medication management. To increase the chances of implementation, it is important to address these individual profession-specific goals and shared team goals [27].

### How do participants appraise and reflect on the new work methods?

Even though this study identified several implementation issues in the home care team, participants seemed to have an all-over positive attitude towards the pharmacist services. Such optimism could serve as a key facilitator in the implementation process. Nevertheless, this favorable view may stem from the increased resources or the personal attributes of the pharmacist, rather than the services provided. The trend could also be explained by the characteristics of early implementation stages in which stakeholders are generally more supportive towards a program. Additionally, the presence of social-desirability bias, where individuals tend to give responses they believe are more favorable or acceptable, may have influenced these positive reports.

Our findings show that even in the absence of any supportive evidence, some participants stated that there was a broad hierarchical consensus that the new practice was “there to stay”. These findings are interesting as they touch upon common yet less scientific approaches to adopting innovations in organizations. Compared to nursing homes and home care settings, Norwegian pharmacists are more commonly integrated in hospital wards. Social network theories describe how healthcare organizations tend to look to organizations of similar size and character when they contemplate whether to take on a new practice or service [39]. In addition, individual “champions” or “experts” can exert great influence on this decision. These networking activities might cause a ‘bandwagon phenomenon’ where effective and less effective interventions spread amongst like-minded organizations. In contrast, implementation science argues that the process of implementing new practices should originate from a solid evidence base, evolve with underpinning program theories, and adapt to context [40, 41].

### Strengths and limitations

The process of integrating pharmacists in multidisciplinary practices outside community pharmacies in Norway is understudied. To the authors’ knowledge, this research is the first to apply implementation theories to assess the embedding of pharmacists in Norwegian primary care. Moreover, it is one of the first to apply the NPT in this context. The study’s theoretical underpinning increases the pragmatic validity of the findings making them valuable in the development of implementation strategies for similar projects.

This study has several limitations. It could be considered a limitation that this study only included a small group of participants from one home care unit. Notwithstanding the seemingly scanty sample size, the participants provide a high degree of information power [42]. Additional participants who could have been recruited include decision-makers and leaders at the higher levels of the local health government, as their insights could have contributed significantly to addressing the study’s objectives.

As pointed out by several of the participants, the period in which the pharmacist services had been provided in the researched setting was relatively short. At the time of data collection, the pharmacists had been employed for approximately 7 months. However, the timing of the research was intentional and in compliance with the study’s aim and objectives. The NPT relates the embedding and normalization of new practices explicitly to the work and efforts involved in the implementation process. Consequently, performing this research at an early stage of integrating the pharmacists in the new setting can provide important information on how to adjust and improve future work.

Implementation theories have limited empirical evidence to support the premises on which they are developed [43]. In the planning of this study, the researchers looked to an extended version of the NPT [23]. By choosing to follow an explicit theory, this study held a preconception that certain events and behaviors are important determinants of the way an intervention can impose the desired changes within an organization. Further, its generic applicability to diverse interventions such as guidelines, diagnostic tools, and collaborative work made it difficult to report equally on every aspect and construct of the theory. Moreover, the theoretical scope of the NPT is narrow and does not consider the effectiveness or quality of a new work method. New practices that are not routinely embedded in everyday practices can still be useful and have value to stakeholders within an organization.

### Generalizability and transferability of the results

This study applies the NPT to investigate how knowledge and behaviors within a home care team can influence the sustainability of newly adopted pharmacist services. We believe our methodology and findings are highly relevant for similar programs. However, determinants of change may arise from different layers and aspects of the context and could be associated with various phases of the implementation process [44]. This complexity makes it difficult to assume that determinants are generalizable.

## Conclusion

Integrating new professions and work methods into clinical practice poses a challenging task that requires implementation skills. The current study conducted a process evaluation of pharmacist integration in a Norwegian home care setting and identified barriers and enablers to the utilization of pharmacist competencies. Our findings emphasize the importance of explicitly defining the collective objectives of pharmacist services in each setting and empowering middle management to drive this process forward. Additionally, clarifying and delineating the scope of practice for each member of the team can mitigate role confusion and intra-professional power struggles.

The evaluation of pharmacist services largely revolves around clinical outcomes and effectiveness. We strongly recommend using implementation theories and frameworks to support the introduction of new practices in healthcare settings. Further, we advocate a focus on process outcomes to better understanding the causal pathways of intervention success and failure.

### Electronic supplementary material

Below is the link to the electronic supplementary material.

Supplementary Material 1

## Data Availability

The datasets used and analyzed during the current study are available from the corresponding author on reasonable request.

## Abbreviations

NPT: Normalization process theory
GP: General practitioner
