# Supplementary material for: Barriers and facilitators of pharmacists’ integration in a multidisciplinary home care team: a qualitative interview study based on the normalization process theory
Source: BMC Health Serv Res. 2024 May 2;24:567. doi: 10.1186/s12913-024-11014-y (PMC11064233; doi:10.1186/s12913-024-11014-y)
Supplement: Supplementary file 1 — Supplementary Material 1 [file 12913_2024_11014_MOESM1_ESM.docx]

**Interview guide**

1. **Introduction/participants' background:**

- Can you tell me a little bit about yourself: age, profession, work experience?

**Research topics:**

**- perception of the pharmacist services (objectives of the pharmacist intervention).**

**- expectations of improved medication work (needs in the setting, available competencies).**

**- perceptions of the implementation process.**

1. **Topic:** **Can you tell me about the pharmacist services in your setting?** **Follow-up questions:**

- How do you perceive the term ‘pharmacist services’?
- Can you tell me why you believe these services are necessary?
- What are the objectives of these services?
  - Can you elaborate on specific activities that the pharmacist performs? What works well?
- Is there a pharmacist work description or pharmacist services SOP?

1. Topic**: Can tell me how you perceive the medication-related challenges in the home care setting? Follow-up questions:**

- What are the most pressing challenges and what activities do the pharmacist have to perform to solve them?
- What competencies do you expect the pharmacist to provide to the team?
- What are your impressions of the new work methods/new services?
- How has medication management improved/changed after the introduction of the new services?
- What are your thoughts on the future of primary care pharmacists and the services they provide?

1. **Topic: Can you tell me how the municipality ensures that the pharmacist and the pharmacist services are optimally utilized in the setting? Follow-up questions:**

- How do you perceive the implementation process?
- Who is responsible for the implementation/who do the pharmacists report to?
- To what degree were you empowered to influence the implementation?
- How do the different healthcare professions collaborate in everyday work?
- What specific activities have been performed to integrate the pharmacists/implement pharmacist services into the collaborative practice?
- Have you experienced any challenges related to the integration of the pharmacist and the pharmacist services?

1. General probes:

- **Can you elaborate on this topic?**
- **Can you give some examples?**
